# Molecular Mimicry: An Uncommon Occurrence of Vitamin B12 Deficiency Imitating Thrombotic Thrombocytopenic Purpura in an African American Male

**DOI:** 10.7759/cureus.45410

**Published:** 2023-09-17

**Authors:** Philipa Owusu-Antwi, Edmund Appiah-Kubi, Vaishali Krishnamoorthy, Evans Takyi, Seetha Murukutla

**Affiliations:** 1 Psychiatry, Richmond University Medical Center, Staten Island, USA; 2 Internal Medicine, Allegheny Health Network, Pittsburgh, USA; 3 Hematology and Oncology, Richmond University Medical Center, Staten Island, USA; 4 Medicine, American University of Antigua, New York, USA

**Keywords:** schistocytes, plasmapheresis, adamts13, vitamin b12 deficiency, thrombotic thrombocytopenic purpura

## Abstract

Thrombotic thrombocytopenic purpura (TTP) and vitamin B12 deficiency can share similar symptoms but require different treatment approaches. TTP is a blood disorder with a high mortality rate requiring immediate plasmapheresis treatment. On the other hand, vitamin B12 deficiency usually presents with anemia, low platelet counts, jaundice, and signs of disrupted red blood cell breakdown, resembling a condition called microangiopathic hemolytic anemia. Vitamin B12 deficiency can sometimes lead to or mimic pseudo-thrombotic microangiopathy (pseudo-TMA), a rare occurrence. Pseudo-TMA manifests as microangiopathic hemolytic anemia and thrombocytopenia and is characterized by schistocytes in a peripheral blood smear.

Differentiating TTP cases from pseudo-TMA cases is essential and should be done promptly. The etiology, treatments, and prognosis of these two conditions differ and can be fatal if not identified and managed. We present a case that emphasizes the need for familiarity with TTP-like conditions, the use of ADAMTS13 as a diagnostic tool, prompt and accurate treatment decision-making, the complexities of therapeutic plasma exchange, and the importance of excluding an enzyme inhibitor or mutator as the cause of TTP or TTP-like cases. Lack of knowledge can lead to erroneous diagnoses, resulting in unnecessary treatments or delayed life-saving interventions.

## Introduction

Vitamin B12, also called cobalamin, derived from the metallic element, possesses a unique molecular structure. It is crucial for DNA synthesis, the production of red blood cells, and the optimal functioning of the neurological system. Cobalamin is an essential cofactor for mutase and methionine synthase enzymes [1]. Individuals suffering from cobalamin deficiency have reduced activity of the methionine synthase enzyme, leading to the inhibition of tetrahydrofolate regeneration. Consequently, the effectiveness of folate is compromised, resulting in impaired DNA synthesis in cells that undergo rapid proliferation. Megaloblastic anemia, cytopenia, and dysplastic changes may manifest in the bone marrow. Megaloblastic anemia-induced hemolysis and ineffective erythropoiesis have been observed to result in the premature depletion of developing red blood cells in the bone marrow [2].

The prompt recognition and assessment of thrombocytopenia and microangiopathic hemolytic anemia (MAHA) in individuals with cobalamin deficiency are crucial due to their potential indication of life-threatening thrombotic microangiopathy (TMA) syndrome. The primary disorders associated with TMA encompass thrombotic thrombocytopenic purpura (TTP), hemolytic uremic syndrome, drug-induced TMA, and complement-mediated TMA. Prompt therapy, such as plasmapheresis or monoclonal antibodies that bind complement proteins, is required to treat these illnesses [3]. Thrombocytopenia and MAHA may also manifest as a consequence of various other medical diseases, including but not limited to severe hypertension, HELLP syndrome, systemic infections, malignancies, autoimmune disorders, and post-solid organ and stem cell transplantation. The focus of the treatment should be directed at addressing the fundamental issue [4-6].

Pseudo-TMA refers to a condition that arises due to an inadequate supply of vitamin B12. Pseudo-TMA symptoms include hemolytic anemia, thrombocytopenia, and dysmorphic *fragmented* red blood cells. People with this condition often experience misdiagnosis with other TMA illnesses and are thus subjected to unnecessary treatments such as plasmapheresis [7]. This report presents a case of pseudo-TMA observed in a patient diagnosed with pernicious anemia and cobalamin deficiency.

## Case presentation

A 45-year-old African American with no past medical history presented to the emergency room with a witnessed syncope. The spouse confirmed loss of consciousness for a few minutes. The patient and his spouse denied tongue biting, nausea, vomiting, posturing, sweating, incontinence, or tremor. He endorsed recent and progressive gait instability, fatigue, tingling/numbness of bilateral lower extremities, loss of balance, decreased loss of sensation in lower extremities, and lower back pain. The family reported witnessing an increasingly confused state and incoherence. They reported the absence of fever, night sweats, early satiety, palpitations, chest pain, or shortness of breath. The patient denied weight loss, nausea, vomiting, constipation, melena, hematuria, and bleeding tendencies. They refuted any precipitating factors or prodromal symptoms such as exertion, strenuous activities (coughing, urination, laughing), emotional distress, crowded places, prolonged sitting or standing, lightheadedness, dizziness, or vertigo. The patient also denied the use of medications. The patient had a family history of colon and prostate cancer; the father was diagnosed at 60 years of age. The patient’s mother also had a history of end-stage renal illness and diabetes. However, the patient denied having any personal or family history of cardiac diseases or blood disorders. The patient did not restrict their diet and ate red meat, although reported a decline in appetite over the preceding few months. Per the patient, he did not follow-up outpatient and had not visited a doctor in years. The patient relayed the social use of alcohol but had not had a drink in months. He quit smoking cigarettes after light use years ago.

Examination showed a fatigued 45-year-old African American obese male with shortness of breath on exertion with lumbar pain but no tenderness on palpation and bilateral lower extremity numbness. All other systems, including cardiovascular, respiratory, abdomen, and neurological, were unremarkable. The patient did not have any empirical findings including reduced strength, power, or sensory perception (including pain and fine touch) on physical examination. There was no observed presence of gait instability. The patient had reflexes within the usual range. The patient’s vital signs were within normal limits; temperature of 98.3°F, heart rate at 76 beats per minute, and blood pressure at 114/64 mmHg (with a mean arterial pressure of 81 mmHg). The respiratory rate was 19 breaths per minute, and the oxygen saturation level was 98% while breathing room air. A head computed tomography (CT) scan revealed the absence of any acute pathological conditions within the cranial cavity. The results of a comprehensive blood analysis indicated the presence of severe macrocytic anemia and thrombocytopenia, as seen in Table 1. The investigation for anemia revealed indications of a deficit in vitamin B12. The peripheral blood smear examination revealed the presence of numerous schistocytes, left shift, and polychromasia. The presence of schistocytes was consistent with MAHA. The laboratory findings showed the presence of transaminitis, an elevation in total bilirubin levels with a predominance of indirect bilirubin, a negative Coombs test result, and normal renal function.

**Table 1:** Labs on admission, hospital course, and at discharge WBC: white blood cell; RBC: red blood cell; Hgb: hemoglobin; Hit: hematocrit; PLT: platelet; PT: prothrombin time; INR: international normalized ratio; APTT: activated partial thromboplastin; LDH: lactate dehydrogenase; AST: aspartate aminotransferase; ALT: alanine transaminase; TIBC: total iron-binding capacity

Lab	On admission (day one) 10:00 am > 11:40 am	Hospital course (day five)	On discharge (day 1)]	Lab reference range
WBC	5.0	3.6	7.4	4–11.2 k/µL
RBC	1.12	2.29	3.05	4.6–6.1 m/µL
Hgb	4.4 > 4.0	7.5	9.6	13.7–17.5 g/dL
Hct	13.2 > 12.4	22.0	29.8	40–51%
Mean corpuscular volume	117.9 > 121.6	96.1	97.7	79–98 fL
Retic count	1.48	-	-	0.9–2.5%
Plt	82 > 76	78	87	150–400 k/µL
Haptoglobin	<8	-	-	43–212 mg/dL
PT	14.4	15.4	-	12–14.8 SEC
INR	1.10	1.21	-	0.9–1.12
APTT	25.7	23.5	-	22.8–36.5 SEC
Fibrinogen	-	209	-	212.1–467.8 mg/dL
D-dimer	-	13.74	-	0–0.52 µg/ml FEU
Total bilirubin	1.5	0.9	-	0.2–1 mg/dL
Direct bilirubin	-	0.4	-	0.0–0.3 mg/dL
LDH	>4,500 (day three)	2,243	684 (day seven)	120–240 U/L
AST	175	29	-	<34
ALT	46	26	-	10–29 U/L
Alkaline phosphatase	31	37	-	120–240 U/L
Vitamin B12	69	-	-	211–911 pg/mL
Methylmalonic acid	-	13,200 (day four)	1,354 (day seven)	87–318 nmol/L
Folate	20.3	-	-	>5.38 ng/mL
Iron	130	-	-	65–175 µg/dL
TIBC	222	-	-	250–450 µg/L
% Saturation	58.5	-	-	20–50%
Transferrin	155	-	-	188–341 mg/dL
Ferritin	470.3	-	-	10.5–307.3 ng/mL
TSH	-	1.384	-	0.55–4.78 µIU/mL

The patient was admitted to the medicine floor for syncope, likely secondary to macrocytic anemia due to B12 deficiency, gastrointestinal bleeding, and thrombocytopenia. The patient underwent a blood transfusion and received a single administration of intramuscular vitamin B12. The team subsequently consulted gastroenterology and hematology. The physical examination was unremarkable upon gastroenterology evaluation except for pale conjunctiva. The differential diagnosis included symptomatic macrocytic anemia, thrombocytopenia, vitamin b12 deficiency, rule-out for colorectal cancer, and atrophic gastritis. The team also had a suspicion of TTP-like syndrome. An abdominal ultrasound examination revealed the presence of splenomegaly, measuring 15.2 cm in length, with normal echogenicity. The patient received daily intramuscular 1,000 µg of vitamin B12 for one week, obtained a hematology evaluation for anemia and thrombocytopenia, and an outpatient referral for endoscopy and colonoscopy. During the hematological assessment, the patient had severe symptomatic anemia, macrocytosis, increasing thrombocytopenia, and mild leukopenia. Repeat analysis of the peripheral blood smear, leukemia, lymphoma panel, coagulation panel, and ADAMTS13 activity was recommended. Therapeutic plasma exchange was also suggested as a treatment option for suspected TTP. In addition, oral prednisone, folic acid supplements, and a continued regimen of intramuscular B12 administration were recommended daily for five days total, followed by weekly. The critical care team came on board for suspected MAHA due to elevated locate dehydrogenase (LDH) (>4,500), decreased haptoglobin (<8), and hyperbilirubinemia, all shown in Table 1, and many schistocytes were seen on peripheral smear. The team recommended transferring the patient to the critical care unit for close monitoring. The patient was determined to require 15 U fresh frozen plasma with each treatment as per the New York blood bank. The plan and treatment were extensively discussed with the patient, who communicated understanding, could teach back, and consented to treatment. A five-day therapy regimen was initiated. During the first session, the patient had pruritus, which was successfully alleviated by administering a single dosage of diphenhydramine. The patient exhibited a varying heart rate of 58 to 64 beats per minute. The patient had no symptoms of lightheadedness, chest pain, palpitations, shortness of breath, or confusion. The electrocardiogram revealed a normal sinus rhythm, with a heart rate of 66 beats per minute and a corrected QT interval (QTc) of 478 ms. The patient’s baseline QTc was 469 ms. The patient exhibited hemodynamic improvement, accompanied by improvements in hemoglobin levels and thrombocytopenia, normalizing coagulation panel, a downward trend in LDH levels, and reductions in total and indirect bilirubin levels. As a result, the patient no longer needed a transfer to the critical care unit. Intrinsic factor antibodies returned positive and ADAMTS13 activity (1.01 IU/mL > 0.79 (0.68-1.63 IU/mL)), complement C3 (103 (82-185 mg/dL)), complement C4 (27 (15-53 mg/dL)), and hepatitis panel were within normal limits. The anti-parietal cell antibody test yielded a positive result with a titer of 1:80, which is higher than the reference range of <1:20. The evaluation for leukemia/lymphoma revealed a slight deviation toward immature white blood cells in the left direction; however, there was no indication of non-Hodgkin lymphoma or acute leukemia.

The patient had persistent asymptomatic sinus bradycardia, particularly during plasma exchange sessions, with fluctuations as low as 44 beats per minute observed on the fourth day of plasma exchange. During this period, the patient also had cognitive disorientation during the plasma exchange process. Electrolytes were optimized. The patient underwent 24-hour telemetry monitoring and echocardiography per the cardiology team’s instructions. The echocardiogram results indicated a left ventricular ejection fraction greater than 80%. A cardiology workup within normal limits suggests the patient’s bradycardic episodes may be related to plasma exchange. The patient received plasma exchange and prednisone treatment for seven days in the hospital. Upon discharge, the patient was prescribed an additional week of prednisone, with a gradual reduction in dosage during outpatient care. Aftercare included hematology and gastrointestinal outpatient follow-ups for further evaluation and management.

## Discussion

**Figure 1:**
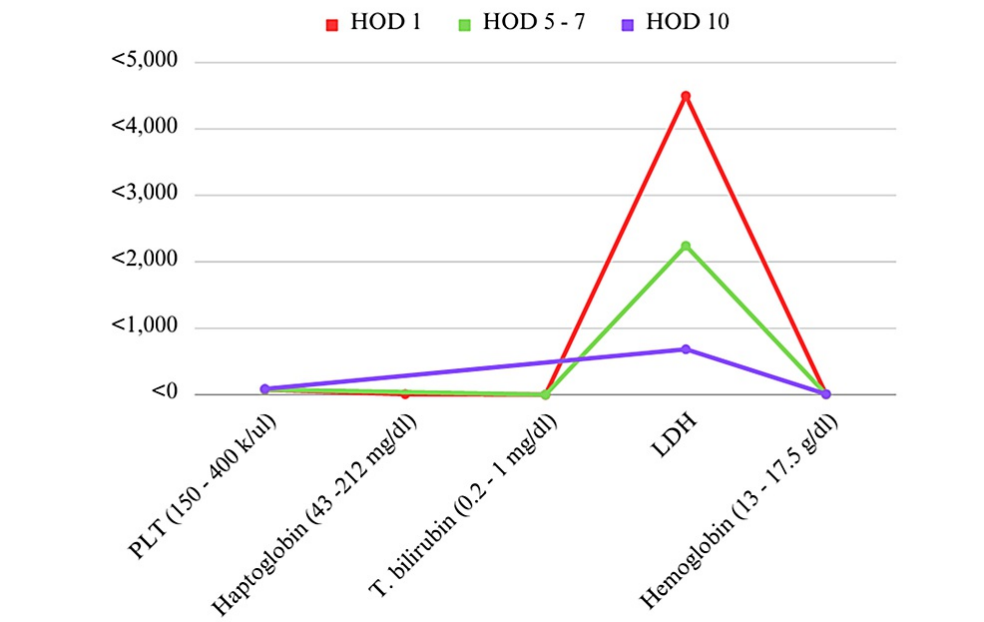
Comparable laboratory symptoms observed in vitamin B12 deficiency and MAHA. MAHA and thrombocytopenia serve as suggestive signs of pseudo-TMA. This figure represents the patient’s laboratory manifestations, such as elevated LDH and reduced haptoglobin on day one of admission, resembling TTP. MAHA: microangiopathic hemolytic anemia; TMA: thrombotic microangiopathy; TTP: thrombotic thrombocytopenic purpura; LDH: lactate dehydrogenase; PLT: platelets; T. bilirubin: total bilirubin; HOD: hospital day The figure was created by Owusu-Antwi.

Pernicious anemia is a form of megaloblastic anemia caused by the autoimmune destruction of parietal cells in the gastric mucosa, leading to impaired absorption of vitamin B12. Primarily, clinical features include macrocytic anemia, but in rare cases, it can present with TMA resembling TTP. Clinically, pseudo-TMA is a rare manifestation [7]. However, due to the similar clinical manifestations of pseudo-TTP and vitamin B12 deficiency, including thrombocytopenia, hemolytic anemia, and neurological abnormalities such as altered sensorium and ataxia, pseudo-TTP may appear to be a common condition. This overlapping presentation can lead to diagnostic confusion and delays in appropriate management. Vitamin B12-induced TMA poses a challenging hurdle for clinicians. The presence of thrombocytopenia and hemolytic anemia threatens the differential diagnosis. The initial evaluation should always seek to rule out life-threatening conditions. Laboratory findings of MAHA, thrombocytopenia, and evidence of end-organ damage suggest TMA. ADAMTS13 activity testing helps differentiate acquired TTP from other causes. TMA develops in about 2.5% of vitamin B12 deficiency cases [8]. The pathogenesis of cobalamin deficiency-induced TMA is poorly understood and may involve homocysteine-induced endothelial injury and dysfunction. Endothelial dysfunction leads to hemolytic anemia from intramedullary hemolysis. Other laboratory evidence includes elevated LDH, indirect hyperbilirubinemia, low haptoglobin, and microangiopathic features on peripheral blood smear. Figure 1 shows the laboratory findings in our patient resembling TTP.

There are some distinguishing features between TTP and cobalamin deficiency-induced TTP-like syndrome. As noted, Noël et al. compared clinical and biochemical parameters in seven patients with cobalamin deficiency-induced TMA and seven with TTP [9]. Seven patients with vitamin B12 deficiency exhibited no acute renal failure, while all those with TTP did. None of the patients with PA-induced TMA also presented with severe neurological symptoms [9]. As with this case report, none of this patient’s classic TTP symptoms were seen. Treatment of TTP involves a multimodal approach, including plasma exchange, corticosteroids, and immunosuppressive therapy. In this case report, our patient’s treatment included plasmapheresis. Although our patient received corticosteroids and plasmapheresis, the concurrent treatment with vitamin B12 injections challenges the response to plasmapheresis, as first noted. Thus, the team cannot conclude if the patient’s positive outcome would have been the same without plasmapheresis.

Utilizing ADAMTS13 as a diagnostic tool

There is a perceived need for an expansion of the existing body of literature and information pertaining to the utilization of ADAMTS13 as a guiding tool for therapeutic plasma exchange. The clinical diagnosis of TTP is supported by the presence of a severe deficit in ADAMTS13. However, it is important to note that ADAMTS13 activity values alone are not sufficient to definitively confirm or exclude the clinical diagnosis of TTP [10]. Hence, the primary determinant for initiating or discontinuing plasma exchange should be the absence or presence of an underlying etiology for the MAHA and thrombocytopenia. The utilization of ADAMTS13 measures is undeniably advantageous; nonetheless, it is important to note that the clinician bears the ultimate responsibility for the management of patients who are believed to have TTP [10,11]. The plasmic score, devised by Pavan Bendapudi, can be used to predict ADAMTS13 deficiency in suspected TTP cases. Even though this test has high discrimination, it can be used to stratify patients according to their need for additional testing and treatment. Previous research has indicated that patients who do not exhibit severe ADAMTS13 deficiency can be effectively managed without an elevated risk of mortality, even in cases where therapeutic plasma exchange is not administered or is discontinued shortly after ADAMTS13 results become available. These findings support the use of ADAMTS13 as a diagnostic tool for TTP and as a means to guide the appropriate administration of therapeutic plasma exchange therapy [12].

Complications of therapeutic plasma exchange

Therapeutic plasma exchange is considered to be a procedure with a reasonably high level of safety as it is associated with minimal occurrence of moderate side effects and problems. The incidence of thrombotic plasma exchange (TPE) problems is impacted by various factors, such as the methods of anticoagulation, the types and quantities of replacement fluid, the vascular access utilized, underlying illnesses, and the techniques employed for plasma separation [13]. The potential adverse effects and problems associated with therapeutic plasma exchange include pruritus, urticaria, hypertension, hypotension, dizziness, nausea, and a sensation of coldness [14]. The presence of hypocalcemia can result in sensory dysfunction in the perioral and finger regions. Replacement therapy for hypocalcemia may lead to hypotension, muscular spasms, headaches, and urticaria. Vascular access placement and anticoagulation are also connected with these symptoms. Additionally, transient vision loss has been reported in relation to these factors [14]. The patient had symptoms of disorientation, bradycardia, and hypernatremia throughout admission, with the presence of confusion and bradycardia being particularly notable during plasma exchange sessions. The observed symptoms may have been a result of the therapy. Hence, it is imperative to conduct further investigation into the association between these symptoms and plasma exchange.

Hypernatremia

The individual described in the case presentation experienced instances of mild hypernatremia, with sodium levels exceeding 145 mmol/L. Patients undergoing therapeutic plasma exchange, similar to the patient described in the case presentation, necessitate vigilant monitoring of their electrolyte levels. A study conducted by Nayak et al. (2020) demonstrated statistically significant alterations in iCa, Na, and K, with a p-value of 0.001 [15]. While electrolyte imbalances generated by TPE may not pose an immediate threat to life and may not necessitate treatment, it is nonetheless recommended to exercise caution and be prepared for potential complications [16].

Bradycardia

Studies have demonstrated the occurrence of cardiac complications, notably arrhythmias, in the context of plasma exchange therapy employed to treat neurological disorders [17]. Bradycardia, characterized by a heart rate below the normal range of 60-100 beats per minute, may be an adverse effect of plasma exchange therapy. Our case report elucidates the possibility that plasma exchange therapy may also induce bradycardia. The occurrence of bradycardia episodes accompanied by transitory loss of consciousness, as observed in the patient presented in our case study, has been identified as a significant complication of plasma exchange therapy [18]. Clinicians must exercise caution, attentively monitor, and swiftly evaluate rare problems that have the potential to result in fatality. The team conducted a thorough and timely investigation of this patient’s episodes of bradycardia to identify any potential underlying or newly developed cardiovascular problems.

Inhibitor versus mutator

A positive result in the intrinsic factor antibody test signifies the presence of autoantibodies that specifically target intrinsic factors within the bloodstream. The presence of megaloblastic anemia, inadequate levels of serum vitamin B12, and a positive intrinsic factor antibody test are highly indicative of pernicious anemia as the underlying pathological state [19,20]. Von Willebrand factor (VWF) is a glycoprotein that is released by vascular endothelial cells in large polymeric structures. It plays a crucial role in facilitating platelet adhesion and aggregation at locations of vascular damage, assuming appropriate ADAMTS13 activity. The process of platelet attachment to VWF is facilitated by elevated levels of shear stress, perhaps due to its impact on the structural arrangement of VWF. The cleavage of VWF is facilitated by ADAMTS13, which is a plasma metalloprotease largely produced by hepatic stellate cells [19]. This cleavage occurs when the flexible macromolecule of VWF is partially unfolded due to elevated levels of shear stress. The protease ADAMTS13 functions to inhibit the buildup of highly active variants of VWF and the aggregation of VWF with platelets by cleaving VWF before its activation by the application of mechanical shear stress. This elucidates the underlying mechanism by which a pronounced shortage in ADAMTS13 leads to the development of microvascular thrombosis, which is a defining feature of TTP. The aforementioned model presents a theoretical structure for understanding the underlying reasons behind the occurrence of microvascular thrombosis in TTP as a consequence of ADAMTS13 deficiency [19].

## Conclusions

Individuals suffering from a pronounced deficit of vitamin B12 may manifest symptoms that are similar to those observed in individuals with TMA syndromes. In the laboratory setting, the identification of high levels of LDH and reticulocyte production can serve as a distinguishing factor between pseudo-TMA and other primary TMA disorders, such as TTP. The utilization of ADAMTS13 as a diagnostic tool in determining the necessity for therapeutic plasma exchange can be considered. It is imperative to promptly and accurately diagnose pseudo-TMA to offer the appropriate treatment involving vitamin B12 supplementation to prevent or minimize the need for needless plasmapheresis therapy. However, one must note that until TTP has been accurately and promptly ruled out, plasmapheresis should be considered as TTP can be fatal. Clinicians must practice prudence and actively monitor and promptly evaluate infrequent complications associated with plasma exchange therapy that can lead to morbidity or mortality.
